# The Humanitarian Lives Saved Tool: An evidence-based approach for reproductive, maternal, newborn, and child health program planning in humanitarian settings

**DOI:** 10.7189/jogh.11.03102

**Published:** 2021-11-27

**Authors:** Victoria B Chou, Angela Stegmuller, Kelsey Vaughan, Paul B Spiegel

**Affiliations:** 1 Institute for International Programs, Department of International Health, Johns Hopkins Bloomberg School of Public Health, Baltimore, Maryland, USA; 2 Bang for Buck Consulting, Amsterdam, Netherlands; 3 Johns Hopkins Center for Humanitarian Health, Johns Hopkins University, Baltimore, Maryland, USA

A shared understanding of key priorities and the path forward is critical to improving reproductive, maternal, newborn, and child health (RMNCH) in humanitarian emergencies. Stakeholders, including local governments, multilateral, United Nations (UN) agencies, and non-governmental organization (NGO) partners, must plan and implement coherent programs to reduce disease burden while working with available financial resources. However, tools to support evidence-based decision making in the challenging context of humanitarian crises are lacking; the paucity of research conducted in a complex humanitarian setting poses additional constraints [1]. The Humanitarian Lives Saved Tool approach (H-LiST) responds to this need, drawing upon humanitarian health, evaluation, costing and modeling principles and evidence about effectiveness of RMNCH interventions from the existing Lives Saved Tool (LiST) model. Here, we present the conceptual framework (**Figure 1**), experiences implementing with in-country partners, and strengths and limitations of the current H-LiST approach. We then discuss gaps and next steps for refining and improving this accessible technical resource.

**Figure 1:**
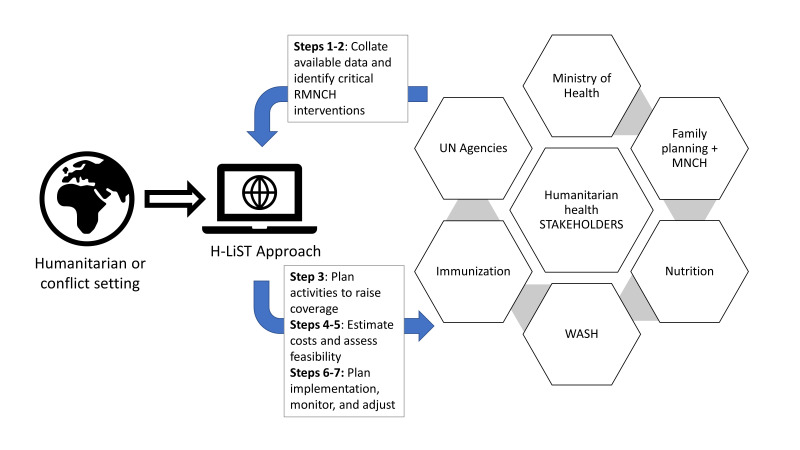
Overview of the Humanitarian Lives Saved Tool (H-LiST) approach.

## THE HUMANITARIAN LIVES SAVED TOOL APPROACH (H-LiST)

Through a seven-step process, the H-LiST approach prompts users to enter key inputs in a guided sequence to ultimately determine which intervention(s) can save the most lives based on current conditions. The rationale for creating the H-LiST platform centered around increasing calls for health-related conflict or disaster responses to adhere to evidence-based principles [2], although evidence about particular interventions in humanitarian crises remains sparse [3]. The H-LiST approach represents a process-driven strategy of knowledge brokering that relies upon consultation and collaboration to engage and build consensus across sectors and actors.

Similar to the LiST model [4], H-LiST is a global health framework based upon the central premise that improving RMNCH outcomes will require expanded delivery of critical interventions in low- and middle-income country (LMIC) humanitarian contexts. Health interventions targeting major causes of maternal or child death in LMICs are grouped together under: pregnancy, childbirth and periconceptional interventions, expanded programme on immunization (EPI), nutrition, outpatient department and community outreach, and water, sanitation and hygiene (WASH). These intervention packages are organized by technical area to reflect traditional delivery by partners with expertise in humanitarian settings.

In the first two steps, H-LiST users collate relevant data from available sources, review the applicability of current indicators, and consider data quality. Given the absence or incomplete nature of data in a humanitarian environment, applying assumptions for certain modeling parameters may be required. Defaults are included for parameters such as age structure and risk factors such as undernutrition but users may also replace these inputs with more relevant data. H-LiST incorporates inputs from the standard LiST model which reflects available data sources of adequate quality representing health conditions at the national level. Aspects of H-LiST can be adjusted if updated or more complete data become available or if differences are noted between improving humanitarian health and RMNCH more generally.

Recognizing that existing and target coverage levels vary across humanitarian settings and technical areas, users select an appropriate scenario range for each technical area to represent the transition from baseline to target intervention coverage (eg, 40% increased to 60%, 60% increased to 80%). Information about intervention effectiveness in LMICs relies on assumptions collated as part of LiST [4]. From a web-based LiST application programming interface (API) [5], users are presented with a ranked list of interventions deemed most important for their context based upon maternal and child lives saved.

Users are prompted in step 3 to consider incremental activities needed to increase coverage of prioritized interventions (eg, health systems strengthening, messaging for demand creation, patient education to improve utilization). In step 4, users identify the incremental costs for these activities to improve coverage. Incremental costs reflect any additional resources needed beyond what is already available and budgeted (eg, existing health facility or employed nursing cadre).

Step 5 walks through a feasibility assessment to explore and consider any implementation-related challenges. Step 6 entails work planning after users have examined costs and implementation-related challenges to make their final selection of activities to improve coverage of the prioritized interventions. Finally, step 7 fosters sustainability as users are encouraged to routinely monitor progress and adjust as necessary to reduce maternal, newborn, and child mortality in a cost-effective way. More information about each step and accompanying worksheets are available on the H-LiST website.

## IN-COUNTRY PARTNER ENGAGEMENT

We conducted a series of workshop sessions to pilot the H-LiST approach in September 2019 in a Ugandan refugee setting. The goal was to assess the tool's feasibility and document implementation challenges. Led by project team members, participants included representatives from UN agencies, NGOs such as Save the Children UK, Oxfam, Action Against Hunger, Water Mission, and other humanitarian partners who engaged through a series of facilitated exercises.

During the sessions, the H-LiST approach was presented, a walk-through of the activities was conducted, and qualitative feedback was gathered. Program managers and implementers reported that H-LiST would be valuable because few evidence-based tools exist for UN and NGO partners to determine annual program priorities. They cited the importance of conducting a robust needs assessment and developing medium- and long-term strategies which incorporate course correction to address concerns in the program cycle proactively. Participants appreciated that people working in different sectors were brought together; participants who typically focus primarily on WASH, for example, interacted and engaged directly with colleagues from finance or maternal and child health, providing a novel opportunity for sectors to jointly examine data, discuss new ideas, and integrate interventions. Overall, participants expressed the need for an approach like H-LiST and were interested in more evidence-based guidance for prioritization, particularly during the program planning cycle.

## STRENGTHS AND LIMITATIONS

Developing a resource that aims to be relevant and accessible for multiple stakeholders can be complicated, although fostering collaboration in this environment remains feasible and worthwhile [6]. Strengths of the H-LiST approach center around the rapid adaptation of an existing, evidence-based tool. Intervention packages and estimates of intervention effectiveness are not drawn from high-income countries but focus on LMICs. The linear and deterministic model promotes reproducibility and transparency because inputs can be collaboratively reviewed and then modified if needed. This contextualization and the fact that all activities, incremental costs, and feasibility are all user-defined means the approach can be used across diverse settings. All seven steps can be completed in a short period of time so it can be easily incorporated as part of the annual planning process.

The platform's limitations are related to the inability to vary all underlying inputs and data gaps around humanitarian-specific interventions as well as intervention effectiveness tested in these settings. Collection of health data during an emergency may be incomplete and routine monitoring and evaluation data collected in more stable, protracted situations may not directly align with assumptions or indicator definitions employed in the Lives Saved Tool. Many H-LiST options were streamlined to avert incompatibility issues. For example, selection for coverage scale-up was restricted to one pattern of change for each intervention category.

**Figure:**
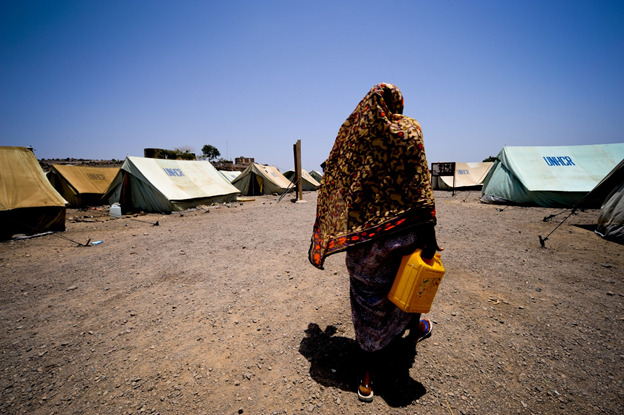
Photo: A woman carries a water container at Al Kharaz Refugee Camp, Yemen. Source: 2009-272. © 2009 Micah Albert, courtesy of Photoshare.

Quality of care is an important factor related to intervention effectiveness but the quality of health systems in humanitarian settings is a dimension that remains poorly understood [7]. H-LiST prioritization of interventions relies upon country-specific profiles for cause of death distributions and baseline mortality rates, but the health status of a local population vs that of vulnerable groups in a conflict setting may differ significantly. Additionally, results from standard LiST are based on national populations which tend to be relatively stable over time. In humanitarian settings, total population may fluctuate widely over the course of a year with unpredictable changes in migration and movement. Coverage trends which are population-based may be difficult to track in a humanitarian context. Due to these constraints, H-LiST was not designed to output the specific number of lives saved, but rather to prioritize those key interventions which have the potential to save the most lives *vis a vis* others.

Lastly, the varying timelines of program implementation by sector, partner, and funding agency can lead to incongruencies which complicate any costing comparison. For this reason, traditional ingredients- or activity-based costing would be inappropriate in this setting. Because health services are often already being delivered and NGOs budget on an annual basis, incremental costing was used to determine what additional infrastructure, materials, personnel, etc., would be necessary to reach target coverage. However, use of incremental costs is problematic in instances where large capital costs are required. For example, WASH interventions incur substantial structural costs at the beginning but lead to great reductions in mortality over time. Partners shared feedback that focusing on incremental costs may discourage NGOs from considering large strategic investments that require commitment up-front but could have a more significant impact over time.

## CONCLUSION

The H-LiST approach is an evidence-based way to assist humanitarian program managers and planners who must determine the most cost-effective interventions to scale up in their context. Led by humanitarian health and evaluation experts, this method was developed through an iterative process with valuable input from various stakeholders including NGOs and UN partners. The model could be improved with better information about the underlying causes of poor RMNCH and the best interventions to address morbidity and mortality during a humanitarian crisis. With calls to bolster the evidence base so that efficient, effective, and sustainable interventions are delivered in these settings [8], the shared goal is to develop a unified vision for progress based upon sound and evidence-based resource allocation. Future versions of the H-LiST approach will ideally be able to provide more nuanced guidance for health program planning in humanitarian settings.

## References

[1] Kohrt BA Mistry AS Anand N Beecroft B Nuwayhid I Health research in humanitarian crises: an urgent global imperative. BMJ Glob Health. 2019;4:e001870. 10.1136/bmjgh-2019-001870 31798999 PMC6861060

[2] Waldman RJ Toole MJ Where is the science in humanitarian health? Lancet. 2017;390:2224-6. 10.1016/S0140-6736(17)31275-8 28602555

[3] Blanchet K Ramesh A Frison S Warren E Hossain M Smith J Evidence on public health interventions in humanitarian crises. Lancet. 2017;390:2287-96. 10.1016/S0140-6736(16)30768-1 28602563

[4] Walker N Tam Y Friberg IK Overview of the Lives Saved Tool (LiST). BMC Public Health. 2013;13 Suppl 3:S1. 10.1186/1471-2458-13-S3-S1 24564438 PMC3847271

[5] Roberton T, McKinnon R. Embedding LiST in a future generation of MNCH modeling tools. Manuscript in preparation.

[6] Leresche E Truppa C Martin C Marnicio A Rossi R Zmeter C Conducting operational research in humanitarian settings: is there a shared path for humanitarians, national public health authorities and academics? Confl Health. 2020;14:25. 10.1186/s13031-020-00280-2 32435274 PMC7222467

[7] Jordan K Lewis TP Roberts B Quality in crisis: a systematic review of the quality of health systems in humanitarian settings. Confl Health. 2021;15:7. 10.1186/s13031-021-00342-z 33531065 PMC7851932

[8] Spiegel PB The humanitarian system is not just broke, but broken: recommendations for future humanitarian action. Lancet. 2017. Online ahead of print. 10.1016/S0140-6736(17)31278-3 28602562

